# Association of heart rate variability, blood pressure variability, and baroreflex sensitivity with gastric motility at rest and during cold pressor test

**Published:** 2021

**Authors:** Kiran Prakash, Amandeep Thakur, Anita S Malhotra

**Affiliations:** *Department of Physiology, Government Medical College and Hospital, Sector 32, Chandigarh, India*

**Keywords:** Electrogastrography, Heart rate variability, Blood pressure, Baroreflex sensitivity, Autonomic nervous system

## Abstract

**Aim:**

To understand the mutual interaction of gastric motility and autonomic functions, the present study evaluated the association of heart rate variability (HRV), blood pressure variability (BPV), and baroreflex sensitivity (BRS) with gastric motility assessed by electrogastrography (EGG) at rest and during CPT and explored the effect of sympathetic activation by cold pressor test (CPT) on gastric motility.

**Background:**

The autonomic nervous system has a significant influence on gastrointestinal motility. HRV is commonly employed to assess the functions of the autonomic nervous system. BPV and BRS are relatively newer techniques and give a more holistic picture of autonomic functions along with the short-term regulation of blood pressure (BP).

**Methods:**

In fourteen young, healthy subjects, gastric motility was assessed by EGG. Beat-to-beat BP and lead II ECG were recorded to assess HRV, BPV, and BRS. BPV and BRS parameters were calculated for systolic, mean, and diastolic BP. Parameters of HRV and BPV were calculated for time and frequency domains. BRS was calculated by sequence and spectral methods.

**Results:**

Significant increases in diastolic BP (*p *= <0.0001) and EGG frequency (*p *= 0.0229) were observed during CPT. Significant correlations were observed between EGG frequencies and many of the HRV, BPV, and BRS parameters. The correlation coefficient was found to be highest between total power of HRV and EGG frequencies during baseline (*p *= 0.0107, r = -0.6571) and during CPT (*p *= 0.0059, r = -0.6935).

**Conclusion:**

EGG frequency can be decreased by an acute increase in sympathetic activity induced by CPT. The novel findings are the significant correlations between many of the HRV, BPV, and BRS parameters and EGG frequency.

## Introduction

 Gastric dysmotility is a basic pathology in many diseases, e.g., irritable bowel syndrome, diabetic gastropathy, inflammatory bowel disease, etc. (1–4). The autonomic nervous system has a significant regulatory effect on gastrointestinal motility (5, 6). Many diseases with altered gastric motility have been reported to be associated with alterations in autonomic nervous functions (7, 8). Few studies have explored the association of autonomic dysfunction with gastric dysmotility, and very few have been carried out on healthy individuals to study the same (9, 10).

Electrogastrography (EGG) is an established and reliable technique for the non-invasive assessment of gastric myoelectrical activity (11, 12). It provides information about slow waves generated and propagated in the stomach in terms of frequency and amplitude (11, 12). It has been observed that sympathetic or parasympathetic activation significantly affects EGG frequency (13–15). Stress- induced autonomic alterations indicated by inhibited vagal activity has been shown to be associated with inhibited gastric myoelectrical activity (16) in healthy subjects.

Heart rate variability (HRV) is a widely accepted method for assessing autonomic function (17). Blood pressure variability (BPV) and baroreflex sensitivity (BRS) are relatively newer and validated tools to assess the arterial baroreflex and autonomic functions (18, 19). These parameters give a holistic view of autonomic function along with the short-term regulation of blood pressure (BP), supplementing the information of HRV. Autonomic dysfunction causes dysregulation of BP; therefore, the assessment of HRV, BPV, and BRS provide deeper and broader information about autonomic and some vascular functions compared to HRV alone (18, 20, 21). To the best of our knowledge and based on a literature search, no study has explored the association of EGG with BPV and BRS.

The cold pressor test (CPT) is an established and validated procedure for assessing the sympathetic function of any individual. CPT has been used in many studies to explore the effect of stress on cardiovascular health (22, 23) and in a few to study the effect of cold stress on gastrointestinal motility (9, 24). In the present study, CPT was carried out to explore the effect of sympathetic activation on the EGG. Studying the effect of autonomic alterations, induced by CPT and quantified by HRV, BPV, and BRS, on gastric motility will help provide a better understanding and aid in exploring the physiology behind the autonomic effect on gastric motility during stress.

To further understand the mutual interaction of gastric motility and autonomic functions, the present study evaluated the association of HRV, BPV, and BRS with gastric motility at rest and during CPT and explored the effect of sympathetic activation by CPT on gastric motility. 

## Methods

Fourteen healthy subjects [9 female, 5 male] were recruited for the present study. Healthy subjects, between the ages of 18 and 35 years were identified after obtaining a detailed clinical history and performing standard clinical physical examinations.

Subjects with a history of any gastrointestinal surgery were excluded from the study. All female subjects were studied during the early follicular stage of their menstrual cycle to minimize possible hormonal effects on the recordings. After obtaining written informed consent from the subjects, they were called on the subsequent day for the recordings. Subjects were asked to report to the Autonomic Function Laboratory, Department of Physiology at Government Medical College and Hospital, Chandigarh, India, after overnight fasting. The present study was approved by the institute’s research and ethics committee. Demographic details were noted before the recordings.

Data acquisition

All recordings were carried out in a room maintained at ambient temperature. Subjects were asked to rest in a lying down position for at least 15 minutes. After ensuring the resting state, the beat-to- beat BP, lead II ECG, and EGG were recorded for thirty minutes. Beat-to-beat BP was recorded non- invasively by the ‘volume clamp’ method of Penaz with finger plethysmography using a Finometer (Finapres Medical Systems, The Netherlands). It gives the waveform of brachial arterial pressure on a beat- to-beat basis. Lead II ECG was recorded using LabChart Pro 7® software (AD Instruments, Australia) at a sampling rate of 1 kHz and a bandpass filter between 0.5 Hz and 35 Hz.

For the EGG recording, the abdominal area of the subjects was thoroughly cleaned with an abrasive sponge and volatile cleanser. Patch electrodes were placed in predefined places (3). Abdominal cutaneous electrical signals were acquired, amplified, filtered, and recorded using LabChart Pro 7® software (AD Instruments, Australia). The sampling rate was kept at 1 kHz with the bandpass filter between 0.016 Hz and 0.3 Hz. Power spectral density analysis of acquired EGG signals was carried out by Fast Fourier Transformation with Hann (cosine-bell) data window. EGG frequency was expressed as cycles per minute (cpm).

For CPT, subjects were asked to put their right hand up to the wrist in cold water (10 ˚C) for one minute. Subjects were instructed to keep their immersed hand open and not touch the walls of the container. Recordings of beat-to-beat BP, Lead II ECG and EGG were carried out at rest, during CPT, and fifteen minutes afterwards.

Analysis of heart rate variability and blood pressure variability

Short-term HRV was analyzed by five-minute lead II ECG recorded with the subjects in a resting state. LabChart Pro 7® software (AD Instruments, Australia) was used to analyze ECG signal and extract RR interval (RRI) data. Nevrokard™ software was used to calculate different parameters of HRV in the time and frequency domains. Among the time domain parameters, variance and coefficient of variance represent the spread of RRI data from the mean. SDNN represents the standard deviation of RRI or the square root of the variance. SENN represents the standard error of RRI. SDSD was calculated as the standard deviation of the differences between adjacent RRI. RMSSD was calculated as the square root of the mean of the sum of the squares of differences between adjacent RRI. NN50 represents the number of those adjacent RRIs which differ by more than 50 ms.

Frequency domain analysis of HRV was carried out by power spectral density analysis of tachograms (graphical representations of RRI data with respect to time) by Fast Fourier Transformation. Power spectral distribution in low (LF; 0.04Hz – 0.15Hz) and high (HF; 0.15 – 0.40Hz) frequency bands was calculated in terms of absolute power and normalized unit. The normalized unit represents the absolute power of the respective band proportional to the total power in the very-low-frequency band. LF/HF ratio was calculated as a ratio of LF-to-HF power.

Similar to the HRV analysis, BP data was pre- processed by LabChart Pro 7® software (AD Instruments, Australia) for BPV analysis. BPV was calculated in the time and frequency domains based on statistical principles similar to those of HRV analysis. All the parameters, such as variance, coefficient of variance, SDNN, SENN, SDSD, RMSSD, absolute and normalized units in LF and HF frequency bands, total power, and LF/HF ratio, were calculated by Nevrokard™ software for systolic, mean, and diastolic BP.

Analysis of baroreflex sensitivity

The pre-processing of beat-to-beat BP and ECG data was carried out by LabChart Pro 7® software (AD Instruments, Australia). BRS was estimated by sequence and spectral methods by Nevrokard™ software. Analysis of BRS by sequence method was carried out by identifying the sequences of three or more consecutive beats characterized by a progressive change in BP (rise or fall) and RRI (lengthening or shortening). The criteria used for identifying sequences were (1) RR variation greater than 5 ms, (2) BP changes greater than 0.5 mmHg, (3) sequences of 3 beats or longer, and (4) sequence correlation coefficient greater than 0.85. BRS by sequence method was estimated by averaging the slope of regression lines identified by the above-mentioned criteria. This analysis was carried out for up- sequences, down-sequences, and all sequences of systolic, diastolic, and mean BP. An additional novel aspect of BRS was estimated by calculating the number of up-, down-, and all sequences based on the above-mentioned criteria during 5 min baseline recordings of beat-to-beat BP and RRI.

To determine BRS by a spectral method, simultaneously recorded beat-to-beat BP and RRI signals were analyzed by fast Fourier transformation. The coherence between power spectral densities of RRI and BP in low frequency (LF, 0.04 – 0.15 Hz) and high frequency (HF, 0.15 – 0.40 Hz) bands was computed. The baroreflex gain was calculated by dividing the amplitude of RR oscillations by the amplitude of corresponding oscillations in systolic, mean, and diastolic BP in the ‘Low’ and ‘High’ frequency bands. These were referred to as α-LF and α-HF in their respective frequency bands. Both α-LF and α-HF are expressed as ms/mmHg. The LF/HF ratio was calculated by dividing the power into low and high-frequency zones of the frequency distribution spectrum of systolic, mean, or diastolic BP.

Statistical analysis

Descriptive statistics were used to summarize all the variables. All parameters were tested for distribution of data using standard normality tests (D’Agostino- Pearson omnibus normality test, Kolmogorov-Smirnov test, and Shapiro-Wilk test). Data with normal distribution are expressed as mean ± SD; data with non- parametric distribution is expressed as median with interquartile range. To compare the mean or median of different parameters during baseline and CPT, the paired t-test or Wilcoxon matched paired test were performed, depending upon the distribution of data.

Depending upon the parametric and non-parametric distribution of data, the association between two parameters was evaluated using Pearson’s or Spearman’s rank correlation coefficient. The levels of statistical significance were set at *p*<0.05. Statistical analyses were carried out using GraphPad Prism version 5.01 for Windows (GraphPad Software, Inc., USA). 

## Results

The present study was conducted on fourteen young, healthy individuals (5 males and 9 females). The baseline demographic characteristics of the subjects are listed in table 1.

**Figure 1:**
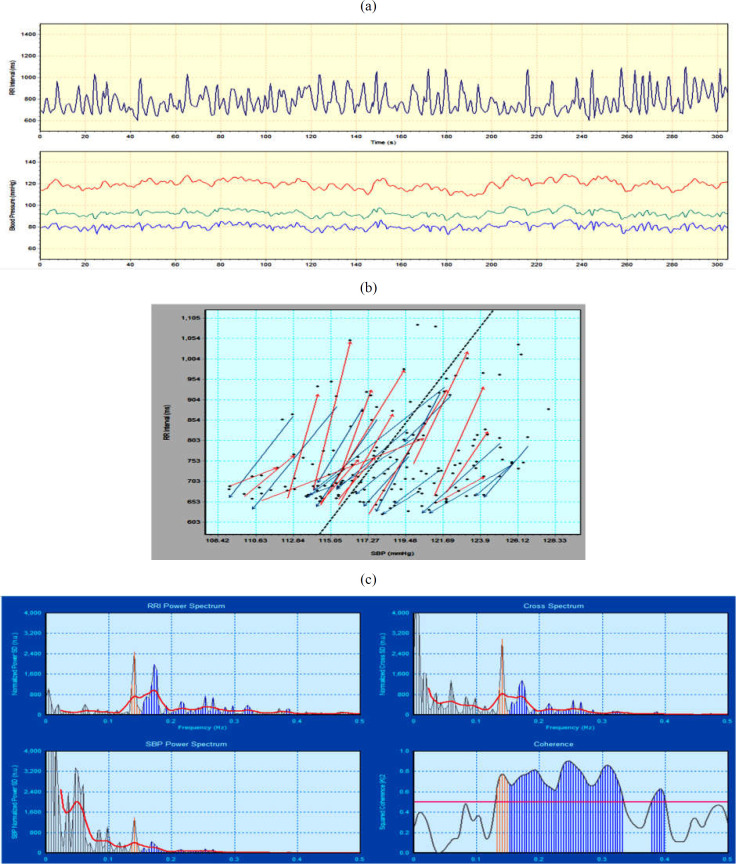
A representative record showing variability of (a) RR interval, systolic blood pressure, mean blood pressure, and diastolic blood pressure; (b) Baroreflex sensitivity by sequence method, where the sequences of three or more consecutive beats are characterized by progressive change in systolic blood pressure and RRI is identified by RR variation greater than 5 ms, blood pressure changes greater than 0.5 mmHg, and sequences correlation coefficient greater than 0.85; and (c) Baroreflex sensitivity by spectral method is shown. SBP, systolic blood pressure

Sympathetic activation by CPT caused significant changes in the EGG frequency, systolic, diastolic, and mean BP, and heart rate (table 2). In addition to a significant decrease in the EGG frequency during CPT as compared to baseline, an increase in its coefficient of variance (0.05 vs 0.20) was found. The values of different time domain and frequency domain parameters of HRV and BPV during baseline are represented in Tables 3 and 4, respectively. The results of BRS analysis by sequence and spectral methods during baseline are shown in Table 5. This table also shows the number of up-, down-, and all sequences between the ramps of beat-to-beat systolic, mean, and diastolic BP, and RRI. A representative record of BRS analysis is shown in Figure 1.

**Table 1:** Demographic details of the subjects

Parameter	Value
Age (years)	29.07 ± 5.36
Weight (Kg)	76.61 ± 18.53
Height (cm)	167.10 ± 9.59
Body mass index (Kg/m^2^)	27.19 ± 4.68
Waist circumference (cm)	87.57 ± 10.88

Normally distributed data is expressed as mean ± SD

**Table 2:** Effect of cold pressor test on different parameters

Parameter	Baseline	During Cold Pressor Test	*p-*value
Systolic blood pressure (mmHg)	118 ± 5.08	141.6 ± 8.78	< 0.0001
Diastolic blood pressure (mmHg)	78.14 ± 5.29	94.86 ± 6.92	< 0.0001
Heart rate (counts per minute)	67.00 ± 6.58	72.00 ± 7.20	0.0093
Electrogastrographic frequency (cycles per minute)	3.40 ± 0.17	3.00 ± 0.59	0.0229

Normally distributed data is expressed as mean ± SD

**Table 3:** Different parameters of heart rate variability during baseline

Time Domain Parameters	
CV	6.75 ± 2.61
VAR	4302 ± 3377
SDNN	61.17 ± 24.55
SENN	3.35 ± 1.44
SDSD	61.76 ± 34.06
RMSSD	61.67 ± 34.01
NN50	33.83 ± 21.17
Frequency Domain Parameters	
LF	1260 ± 1157
LF (nu)	42.28 ± 16.22
HF	953.90 (430.2-2613)
HF (nu)	50.63 ± 16.23
TP	2743 (1873-5301)
LF/HF	0.76 (0.56-1.64)

Normally distributed data is are expressed as mean ± SD, and non-parametric data is expressed as median (inter-quartile range). CV, coefficient of variance; VAR, variance; SDNN, standard deviation of RRI or square root of the variance; SENN, standard error of RRI; SDSD, standard deviation of the differences between adjacent RRI; RMSSD, square root of the mean of the sum of the squares of differences between adjacent RRI; NN50, number of those adjacent RRIs which differ by more than 50ms; LF, absolute power in low-frequency band; nu, normalized unit; HF, absolute power in high-frequency band; TP, total power; LF/HF, ratio of LF-to-HF power

**Table 4:** Different parameters of blood pressure variability during baseline

Time Domain			
Parameters	SBP	MBP	DBP
CV	2.87 ± 0.84	2.20 ± 0.62	2.24 ± 0.62
VAR	12.16 ± 6.58	4.42 ± 2.49	3.55 ± 1.91
SDNN	3.36 ± 0.96	2.03 ± 0.56	1.83 ± 0.49
SENN	0.18 ± 0.06	0.11 ± 0.03	0.10 ± 0.03
SDSD	1.68 ± 0.48	0.96 ± 0.25	1.14 ± 0.37
RMSSD	1.17 ± 0.48	0.96 ± 0.25	1.14 ± 0.37
Frequency Domain			
Parameters	SBP	MBP	DBP
LF	27.84 ± 14.94	16.10 ± 6.99	13.50 ± 5.82
LF (nu)	67.38 ± 20.11	79.62 ± 12.03	71.71 ± 11.22
HF	11.29 ± 8.70	3.15 ± 2.51	2.67 (1.79-6.74)
HF (nu)	27.75 ± 19.71	15.63 ± 11.30	21.87 ± 10.21
TP	127 ± 62.11	45.73 ± 24.12	36.64 ± 18.30
LF/HF	5.16 ± 5.41	8.29 ± 5.81	3.74 (2.20-7.60)

Normally distributed data is expressed as mean ± SD, and non-parametric data is expressed as median (inter-quartile range). SBP, systolic blood pressure; MBP, mean blood pressure; DBP, diastolic blood pressure; CV, coefficient of variance; VAR, variance; SDNN, standard deviation of beat-to-beat BP or square root of the variance; SENN, standard error of beat-to-beat BP; SDSD, standard deviation of the differences between adjacent beat-to-beat BP; RMSSD, square root of the mean of the sum of the squares of differences between adjacent beat-to-beat BP; LF, absolute power in low-frequency band; nu, normalized unit; HF, absolute power in high-frequency band; TP, total power; LF/HF, ratio of LF-to-HF power.

**Table 5:** Different parameters of baroreflex sensitivity during baseline

BRS Parameters	SBP	MBP	DBP
Sequential BRS accessed by UP sequences of BP and RR interval	31.26 (21.21 -.42.91)	49.62 ± 30.96	45.31 ± 19.43
Sequential BRS accessed by DOWN sequences of BP and RR interval	26.68 ± 15.27	42.57 ± 22.37	41.60 ± 22.24
Sequential BRS accessed by ALL sequences of BP and RR interval	37.81 ± 22.43	47.03 ± 25.84	44.97 ± 18.59
αLF of spectral BRS	8.33 ± 3.97	9.71 ± 4.26	10.33 ± 4.36
αHF of spectral BRS	12.70 ± 6.52	22.19 ± 14.05	18.78 ± 5.14
Number of UP-sequences of BP and RR interval	8.07 ± 6.69	5 (3.75-9.50)	9.79 ± 6.62
Number of DOWN-sequences of BP and RR interval	5 (1.00 - 7.75)	4 (3.0 - 8.5)	6.64 ± 5.39
Number of ALL sequences of BP and RR interval	14.50 ±11.67	9 (7.0 - 18.5)	16.43 ± 10.85

Normally distributed data is expressed as mean ± SD, and non-parametric data is expressed as median (inter-quartile range). BRS, baroreflex sensitivity; BP, blood pressure; αLF, α coefficient in low-frequency band; αHF, α coefficient in high-frequency band.

**Figure 2:**
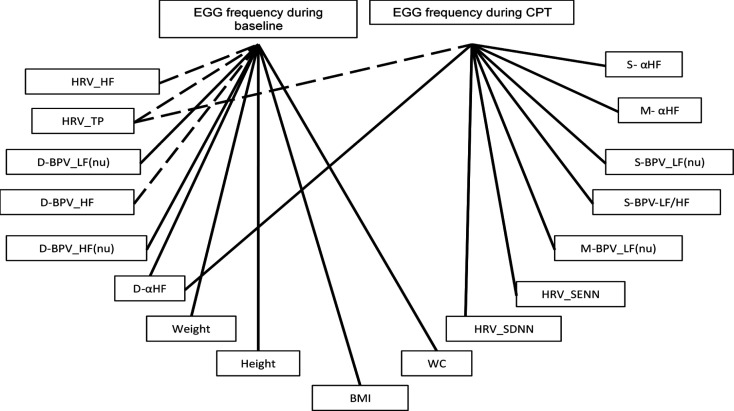
Correlation web showing significant correlations between EGG frequencies (during baseline and during CPT) and various HRV, BPV, and baroreflex sensitivity parameters. EGG, electrogastrography; CPT, cold pressor test; HRV, heart rate variability; BPV, blood pressure variability; HF, absolute power in high-frequency band; TP, total power; D, diastolic; LF(nu), power in normalized unit in low-frequency band; HF(nu), power in normalized unit in high-frequency band; αHF, α coefficient in high-frequency band; BMI, body mass index; WC, waist circumference; SDNN, standard deviation of the differences between adjacent waveform; SENN, standard error of data; M, mean; S, systolic; LF/HF, ratio of LF-to-HF power

Correlation analysis was carried out between EGG frequency and different anthropometric, HRV BPV, and BRS parameters (Figure 2). Significant correlations were found between EGG frequency during baseline and HF (p= 0.0181, r= -0.6196) and total power (p= 0.0107, r= -0.6571) of HRV; LF (nu) (p= 0.0342, r= 0.5677), HF (p= 0.0483, r= -0.5358), and HF(nu) (p= 0.0237, r= -0.5986) of diastolic BPV; αHF (p= 0.0155, r= -0.6313) of BRS derived from spectral analysis of diastolic BP and RRI; weight (p=0.0046, r= 0.7086), height (p= 0.0398, r= 0.5541),

body mass index (p= 0.0264, r= 0.5898), and waist circumference (p= 0.0272, r= 0.5875). Moreover, significant correlations were found between EGG frequency during CPT and SDNN (p= 0.0357, r= -0.5639), SENN (p= 0.0281, r= -0.5847), and total power (p= 0.0059, r= -0.6935) of HRV; LF (nu) (p= 0.0471, r= -0.5382) and LF/HF (p= 0.0067, r= -0.6865) of systolic BPV; LF (nu) of mean BPV (p= 0.0465, r= -0.5395); and, αHF of BRS derived from spectral analysis of systolic, mean and diastolic BP and RRI (p= 0.005, r= -0.7036; p= 0.0073, r= -0.6811; p= 0.0404, r= -0.5526, respectively).

## Discussion

The major novel finding of the present study is the significant correlation between EGG frequency and various BPV and BRS parameters along with HRV. The present study also reports a significant decrease in EGG frequency during CPT as compared to baseline. This implies that even the transient acute increase in sympathetic activity may cause an increase in gastric myoelectrical activity. Similar findings have been reported by Fone et al. (9), who observed a significant decrease in the number of antral and propagated antroduodenal pressure waves after cold stress.

BRS assesses the sensitivity of arterial baroreflex loop or baroreceptor – heart rate reflex, which plays a significant role in maintaining BP homeostasis (18, 20). Impaired BRS has been observed in many diseases (18–21, 25). To the best of our knowledge and according to our literature search, no study has assessed BPV and BRS in any gastrointestinal disease. Many methods have been employed to determine BRS in the literature. A relatively newer and widely accepted technique is the assessment of spontaneous baroreflex control of heart rate by sequence and spectral methods (18, 26). The benefit of these techniques is that they do not require any external intervention or any effort by the subject. The present study has shown a significant negative correlation with the EGG frequencies during baseline and CPT. None of the BRS parameters, assessed by sequence method, showed a significant correlation with EGG frequencies; however, αHF calculated by spectral analysis of BP (systolic, mean, and diastolic) and RRI showed a significant correlation with EGG frequency during CPT. Coherence between BP and RRI in high-frequency bands have some contribution from non-baroreflex mechanisms in addition to the baroreflex loop. αHF also has some contribution from respiratory rate and hormonal influence. It can be speculated that the non-baroreflex mechanisms which affect αHF also affect EGG frequencies, rather than the baroreflex loop per se (26, 27). However, further detailed studies are required to decipher the underlying mechanisms of the associations between BRS and EGG.

As the autonomic nervous system has a strong regulatory effect on gastrointestinal motility (5, 6), significant correlations between EGG frequencies and various autonomic parameters assessed by BP and HRV can be expected. Among the HRV parameters, HF and total power were found to be significantly correlated with EGG frequency during baseline. HF and total power of HRV reflected parasympathetic activity (17). Similarly, EGG frequency during CPT was also found to be significantly correlated with HRV parameters, such as SDNN, SENN, and total power, which reflects the parasympathetic activity and sympathovagal balance of the autonomic nervous system (17). A study by Pietraszek et al. (28) showed that EGG and HRV parameters are associated even at the baseline. A study by Neild et al. (29) also highlighted the fact that patients with HIV have significantly decreased gastric emptying and HF power as compared to the control group.

The information provided by the basic hemodynamic parameters and HRV has some limitations, and BPV and BRS provide more holistic information about the function of the autonomic nervous system and cardiovascular health. The arterial baroreceptor reflex system maintains a relatively constant arterial BP and prevents its short-term wide fluctuations. Homeostatic mechanisms of the body aim to keep the systemic BP relatively unaltered as compared to the heart rate (17, 19). Some studies have reported reduced HRV and increased BPV in autonomic dysfunction (30–33). The present study found significant correlations between EGG frequencies during baseline and CPT and various BPV parameters. These results indicate that factors governing BP may affect gastric motility, too. The autonomic nervous system plays a role in BP regulation as well as in regulating gastric motility. Most of the BPV parameters showed significant negative correlations with EGG frequencies. With these observations, it can be hypothesized that the factors which increase BPV or tend to alter the homeostasis of BP decrease gastric motility. This decrease in gastric motility maybe because of the sympathetic activation, which tends to bring down the perturbed systems toward normalcy.

Caution, however, is warranted before generalizing the findings of the present study. Further studies on different diseases and age ranges need to be performed to explore the dependency or autonomy and the extent of gastric motility being affected by ANS. Better imaging techniques and invasive recording of gastric myoelectrical activity may be more valid tools to study gastric motility.

In conclusion, the present study addresses the relationship between EGG and HRV, BPV and BRS, and provides evidence for the fact that even an acute transient increase in sympathetic activity, induced by CPT, can significantly decrease EGG frequency. The novel finding of the present study is the significant correlations between many HRV, BPV, and BRS parameters and gastric motility. However, further studies are required to validate this correlation and use these parameters as predictors of gastric motility.

## Funding

The author(s) received no financial support for the research, authorship, and/or publication of this article.

## Conflict of interests

The authors declare that they have no conflict of interest.
